# A familial t(4;8) translocation segregates with epilepsy and migraine with aura

**DOI:** 10.1002/acn3.51040

**Published:** 2020-04-21

**Authors:** Milena Crippa, Paola Malatesta, Maria Teresa Bonati, Francesco Trapasso, Francesco Fortunato, Grazia Annesi, Lidia Larizza, Angelo Labate, Palma Finelli, Nicola Perrotti, Antonio Gambardella

**Affiliations:** ^1^ Research Laboratory of Medical Cytogenetics and Molecular Genetics IRCCS Istituto Auxologico Italiano Milan Italy; ^2^ Department of Medical Biotechnology and Translational Medicine University of Milan Milan Italy; ^3^ Institute of Medical Genetics University Magna Græcia Catanzaro Italy; ^4^ Clinic of Medical Genetics IRCCS Istituto Auxologico Italiano Milan Italy; ^5^ Institute of Neurology University Magna Græcia Catanzaro Italy; ^6^ Institute of Molecular Bioimaging and Physiology National Research Council Catanzaro Italy

## Abstract

Three relatives carrying a t(4;8)(p15.2;p23.2) translocation had juvenile myoclonic epilepsy, self‐limited photosensitive occipital epilepsy and migraine with aura. The t(4;8) translocation interrupted the coding sequence of *CSMD1* gene and occurred immediately to the 3’UTR of *STIM2* gene. *STIM2* was overexpressed in the patient carrying the unbalanced translocation, and all three individuals had a single functional copy of *CSMD1*. Array CGH study disclosed that these three individuals also carried a deletion at 5q12.3 that involves the *RGS7BP* gene. The overall results favor the view that *CSMD1*, *STIM2,* and *RGS7BP* genes could contribute to epilepsy and migraine phenotypes in our family.

## Introduction

Juvenile myoclonic epilepsy (JME) is inherited as a multifactorial trait,^1^ and it may share genetic determinants with self‐limited photosensitive childhood occipital epilepsy (pCOE), with phenotypic overlap between these two epilepsy syndromes.^2^ There is also a shared genetic susceptibility between these epilepsy syndromes and migraine with aura (MA).^3^ All these findings have been fueled by the description of clearly genetic syndromes in which epilepsy and MA both occur in the same family,^4^, ^5^ as the result of the higher cortical excitability shared by both conditions.^5^

Here, we present a family carrying a t(4;8)(p15.2;p23.2) translocation that segregates with JME, pCOE, and MA phenotypes. We performed a thorough characterization of translocation and breakpoint mapping in affected carriers, which are crucial for positioning disease‐candidate genes at or near the breakpoints. Moreover, high resolution array CGH was applied to unveil other genes possibly related to JME, pCOE, and MA.

## Methods

### Patients and family

**Figure 1:**
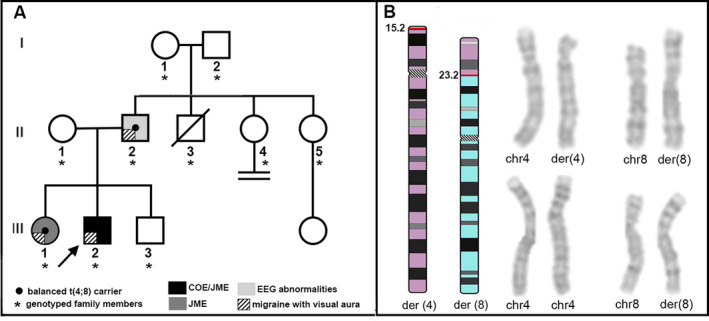
(A) Pedigree of the family. The proband, carrier of the unbalanced reciprocal translocation (derivative of chromosome 8), indicated by the arrow, is depicted as a filled symbol to show he is affected by COE/JME. His father and sister, carriers of the balanced reciprocal translocation, shown as a small circle in the middle of the gender symbols, are filled with different gray degree intensity to signify they are respectively affected by EEG abnormalities the father (light gray) and by JME the sister (dark gray). A diagonal black and white striped quarter‐filled symbol indicates migraine with visual aura. Members genotyped in the study are asterisked. (B) Ideograms illustrating the derivative chromosomes involved in the t(4;8)(p15.2;p23.2) translocation (right), high‐resolution chromosomal GTG banding detecting the unbalanced translocation in the proband (top left) and the balanced rearrangement in his father (down left).

The family (Fig. 1A) originates from Southern Italy. All family members underwent a comprehensive clinical evaluation. In all subjects, the diagnosis of epilepsy was based according to the ILAE criteria,^6^ and the headache diagnosis was established according to standardized criteria.^7^ Blood samples were collected after signature of an informed consent. The study was approved by the Ethics Committee of IRCCS Istituto Auxologico Italiano Milano, Italy, and Regional Ethics Committee, Calabria, Italy.

The electronical features of the family are reported in detail as Data S1. The 22‐year‐old proband (III:2) had childhood pCOE and later developed JME. He also had learning difficulties and aggressive behavior with facial dysmorphisms. Interictal awake and sleep EEGs showed bilateral occipital sharp waves and generalized spikes associated with both generalized and occipital photic‐induced spikes. Lamotrigine (300 mg/day) led to complete control of seizures, and last awake and sleep EEG recording was normal. His 24‐year‐old sister (III:1) had JME that started during adolescence; EEG revealed generalized 3.5‐5 Hz spike–wave discharges and a generalized photo‐paroxysmal response. Levetiracetam was effective. The 48‐year‐old father (II:2) had no seizures, but EEG showed generalized and occipital sharp waves. Since adolescence, all three relatives had MA. The proband’s mother (II‐1) and 19‐year‐old brother (III‐3) did not experience any seizures or MA, and their EEG and brain MRI were normal.

### Genetic study

GTG banding was performed according to standard procedures. aCGH analysis was performed using the Human Genome CGH Microarray Kit 2x400K (Agilent Technologies, Palo Alto, CA), following the manufacturer’s instructions. FISH mapping of the breakpoints (bkps) was performed using BAC clones, targeting the 4p15.2 and 8p23.2 bkp regions.

Specific primers pairs were used to verify the expression of *STIM2*, *CSMD1*, and *RGS7* on control blood RNA. RNA collection and quantitative real‐time RT‐PCR based on the TaqMan methodology, were performed as previously described.^8^ All assays were provided by Thermo Fisher Scientific (TaqMan Gene Expression Assays: ID# Hs00372705_m1 STIM2; Hs99999905_m1 GAPDH; Hs99999910_m1 TBP). For data analysis, we established the proper range of *STIM2* gene expression in 10 healthy controls calculating the mean value ± 2 standard deviation (SD).

Sanger sequencing of the following epilepsy genes was performed: *EFHC1*,*CACNA1A*,*CACNA1H*,*CHRNA4*,*CHRNA7*,*CHRNB2*,*DEPDC5*,*GABRA1*,*GABRD*, *GABRG2*,*KCNQ2*,*KCNQ3*,*KCNT1*,*LGI1*,*PRRT2*,*SCN1A*,*SCN1B*,*SCN2A*,*SCN8A*,*SCN9A, SLC2A1*. Primers list is available upon request.

## Results

Cytogenetic analysis of the proband revealed a paternal origin unbalanced translocation with partial trisomy of chromosome 4 and partial monosomy of chromosome 8: 46,XY,der(8)t(4;8)(p15.2;p23.2)pat (Fig. 1). His father and sister were found to be carriers of the apparently balanced translocation, while the mother (II‐1) and brother (III‐3) had a normal karyotype. The karyotypes of paternal grandfather, paternal aunts II‐4, and II‐5 were normal, while that of paternal grandmother was 47, XXX. In the proband and her sister, no mutation of the epilepsy genes was found.

**Figure 2:**
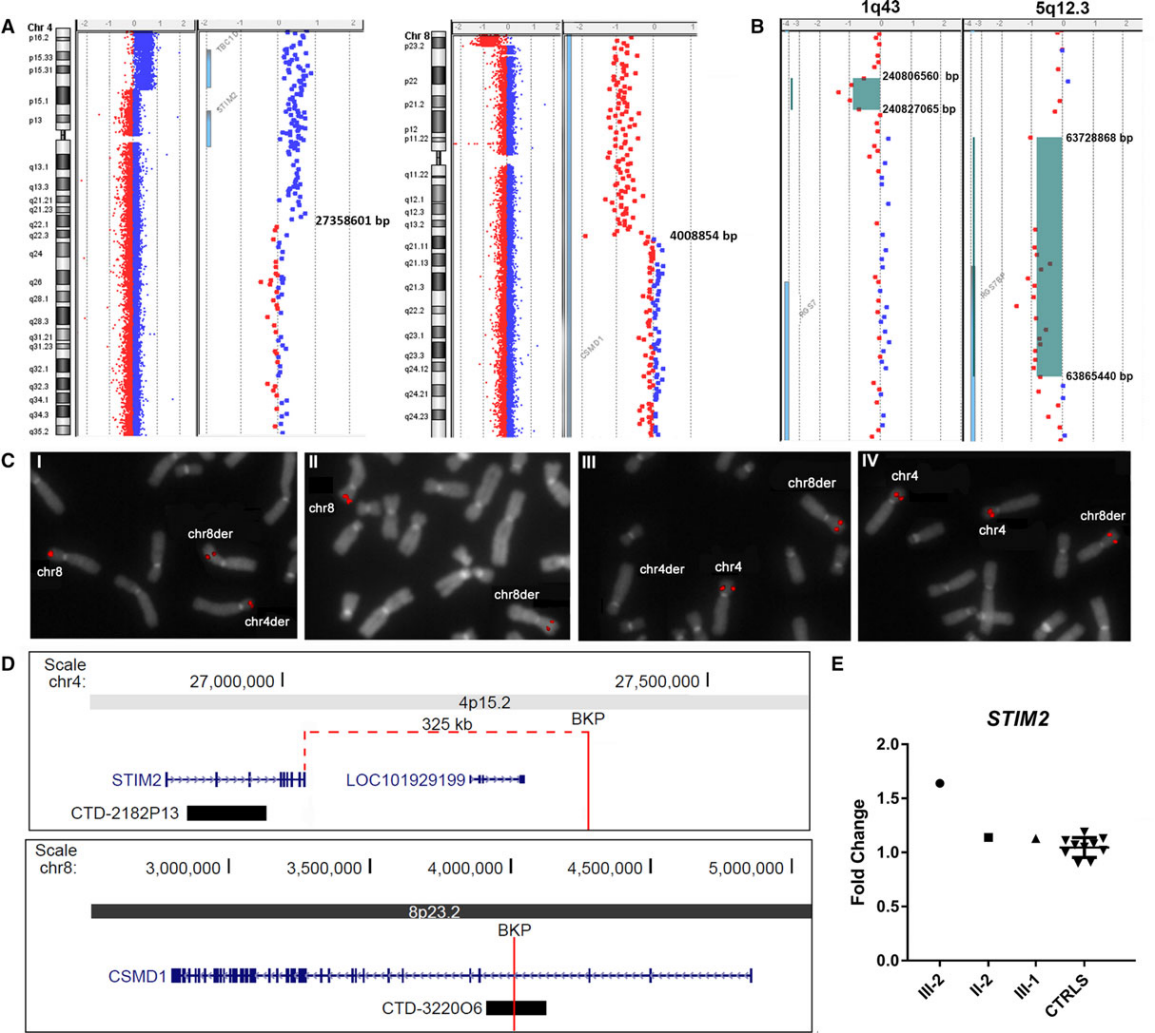
(A) Array‐CGH ratio profiles of proband III‐2. Chromosomes 4 and 8 ideograms showing the 27.3 Mb 4p terminal duplication, the 3.8 Mb 8p terminal deletion, and magnifications of the breakpoint regions showing gene content and genomic positions, according to the genome build CGRh37/hg19. (B) Array‐CGH profiles of two rare CNVs identified in III‐1 genome. 1q43 deletion, identified in III‐1 and her mother (II‐1), located about 112 kb from the 3’UTR of *RGS7* gene, and 5q12.3 deletion, partially involving the *RGS7BP* gene, identified in III‐1, III‐2, and their father (II‐2). Gene content and genomic positions are shown, according to the genome build CGRh37/hg19. (C) FISH analysis of translocation breakpoints. FISH with BAC clone CTD‐322006 (I,II), which spans the translocation bkp at 8p23.3, yields hybridization signal on chromosome 8 and signals of diminished intensity on both derivative chromosomes in II‐2 (I), while in III‐2 (II) the probe produces a signal on chromosome 8 and a signal of diminished intensity on der(8). FISH with BAC clone CTD‐2182P13 (III,IV), partially covering the *STIM2* gene sequence at 4p15.2, yields hybridization signals of equal intensity on chromosomes 4 and der(8) in II‐2 (III), but produces three signals of the same intensity on both chromosomes 4 and on der(8) in the proband III‐2 (IV). (D) Physical map of the genomic regions involved in the bkps. 4p15.2‐15.1 region showing the mapped genes, the position of the 4p translocation bkp and its distance from the 3’UTR of *STIM2* gene, and the probe CTD‐2182P13used for FISH analysis. 8p23.2 region showing the position of the 8p translocation bkp disrupting the *CSMD1* gene at IVS3 and the probe CTD‐322006 used for FISH analysis. (E)* STIM2* gene expression analysis**.** Relative expression of *STIM2* blood mRNA in the proband (III‐2), his father (II‐2), and his sister (III‐1), compared to 10 healthy controls. *STIM2* showed a significant overexpression in III‐2, while a normal expression level was found in II‐2 and III‐1 compared to controls. Data were normalized against *TBP* as housekeeping gene; similar results were obtained using *GAPDH* as normalizer (data not shown). [Corrections added on 21 May 2020 after first publication: Figure 2 has been corrected.]

In the proband, the aCGH analysis identified a 27.3 Mb 4p terminal duplication and a 3.8 Mb 8p terminal deletion (Fig. 2). In detail, the translocation bkps occurred within a region of about 33.8 kb (chr4:27358601‐27392379, hg19), and 2.9 kb (chr8:4008854‐4011844, hg19), respectively. According to the UCSC Genome Browser database, no genes are disrupted at the 4p bkp which maps about 325 kb proximally to the 3’ UTR of the *Stromal interaction molecule 2* (*STIM2*) gene (OMIM*610841), while the 8p bkp falls within intron 3 of the *Cub and sushi multiple domains 1* (*CSMD1*) gene (OMIM*608397) (transcript NM_033225.5) (Fig. 2).

The aCGH analysis confirmed that the translocation in the father and sister was balanced. Furthermore, two rare CNVs localized on chromosomes 1 and 5 were detected in the sister, namely a deletion of 20.5 kb at 1q43 (chr1:240806560‐240827065, hg19) inherited from the mother and a deletion of about 136.6 kb at 5q12.3 (chr5:63728868‐63865440, hg19) inherited from the father (Fig. 2). Both CNVs were not identified in the proband’s brother III‐3, while only the 5q12.3 deletion was present in the proband’s genome. According to the UCSC Genome Browser database, the 5q12.3 deletion partially involves the *Regulator of G‐protein signaling 7 binding protein* (*RGS7BP*) gene (OMIM*610890) (5’UTR, exons 1 and 2, transcript NM_001029875.2), while no coding genes are included in the 1q43 deletion despite the 3’UTR of *RGS7* gene maps 112 kb from the distal deletion bkp.

FISH analyses with BAC probe CTD‐322006 (chr8:3912262‐4127238, hg19), mapping within the IVS3 of *CSMD1* gene, confirmed its disruption by the translocation. The probe spans the 8p translocation bkp and in II‐2 and III‐1 metaphases produced signals on chromosome 8 and on both derivative chromosomes (Fig. 2). In III‐2, CTD‐322006 yielded a signal on chromosome 8 and a signal of diminished intensity on der(8) (Fig. 2). FISH with BAC probe CTD‐2182P13 (chr4:26888710‐26981066, hg19), partially covering the *STIM2* gene sequence, showed equal signals on chromosomes 4 and der(8) in II‐2 and III‐1, but produced three signals of the same intensity on both chromosomes 4 and on der(8) in the proband III‐2 (Fig. 2).

The expression level of *STIM2*, *CSMD1*, and *RGS7* genes, was analyzed on II‐2, III‐1, and III‐2 blood RNAs. *STIM2* showed a significant overexpression in III‐2, who has three copies of the gene, compared to controls (Fig. 2), while in II‐2 and III‐1 the *STIM2* gene expression level was comparable to controls (Fig. 2). Both *CSMD1* and *RGS7* mRNA were not quantifiable in blood.

## Discussion

Our family emphasizes the electro‐clinical overlap between JME and pCOE,^2^ and their pathophysiological link with MA, with coexistence of both epilepsy and migraine phenotypes in the same family.^4^, ^5^ The affected members were found to carry a t(4;8)(p15.2;p23.2) translocation that interrupted the coding sequence of *CSMD1* at 8p23.2 and occurred at 4p15.2 nearby the 3’UTR of *STIM2* gene. Array CGH study also disclosed that the three affected individuals carried a rare deletion at 5q12.3 that partially involves the *RGS7BP* gene, while mutations in genes mostly associated with both epilepsy and migraine were ruled out. Thus, considering the oligogenic origin of epilepsies,^6^ it is reasonable to speculate that the detrimental effect of the rearrangement on *CSMD1* and hypothetically *STIM2*, together with *RGS7BP* deletion may contribute to the epilepsy/migraine phenotypes in our family.

The three affected relatives, indeed, carried a single functional copy of *CSMD1*, which is highly expressed in the brain, especially in regions of neuronal differentiation and outgrowth, consistent with a role in synaptic pruning and synaptic plasticity.^9^ Accordingly, CSMD1 has a broad role in neurodevelopment illness phenotypes and is also similar to *CSMD3*, located at 8q23, already associated with a similar phenotype of benign adult familial myoclonic epilepsy.^10^ Moreover, a partial duplication of *CSMD1* was associated with myoclonic seizures, developmental delay and autism in a child,^11^ and isolated *CSMD1* deletions contributed to the epilepsy phenotype.^12^

*STIM2*, likewise *STIM1,* encodes for a protein sensor for luminal calcium that modulates the activity of membrane channels, thus regulating calcium conducting channels and sensors.^13^ Accordingly, the expression of both *STIM1* and *STIM2* is highly increased in hippocampal specimens of a patient with temporal lobe epilepsy.^14^ In our family, *STIM2* might contribute to the disease phenotype in the proband, as he bears three copies of *STIM2* leading to its overexpression in blood. In the other two relatives, however, we did not observe any difference compared to controls in blood, making its contribution more elusive. The *RGS7BP* gene, deleted in all affected members, may also contribute to the phenotype in our family. Indeed, it is a dosage sensitive gene (pLI = 1), highly expressed in the brain, encoding a membrane protein that binds and regulates all members of the R7 subfamily of regulators of G protein signaling, including the RGS7 protein, forming a complex involved in the regulation of nervous system development and function.^15^

Lastly, the more complex phenotype of the proband is likely explained by the double segmental aneuploidy, resulting from the unbalance translocation, as similar clinical features were already associated with both pure 8pter deletions and 4pter duplications.^16^, ^17^ Because of these complex clinical findings, the diagnosis of pCOE in the proband might be questioned, but it is well recognized that self‐limited focal epilepsy may occur in children with abnormal neurological examination and, even, known brain injury.^18^

In conclusion, our study provides novel evidence that *CSMD1, STIM2,* and *RGS7BP* might be candidates to contribute to JME, pCOE, and MA in our family. Further studies are warranted to better define their role in the pathogenesis of these clinical phenotypes.

We confirm that we have read the Journal’s position on issues involved in ethical publication and affirm that this report is consistent with those guidelines.

## Author Contributors

Drs Crippa, Malatesta, Finelli, and Gambardella contributed to design, conceptualization of the study, generation and interpretation of the data, and revising the manuscript. Drs Annesi, Bonati, Trapasso, and Fortunato contributed to the generation and interpretation of the data and drafting the manuscript. Drs Labate, Larizza, and Perrotti contributed to conceptualization of the study, and revising the manuscript.

## Supporting information


**Data S1.** Clinical features of family members.
