# Supplementary material for: A familial t(4;8) translocation segregates with epilepsy and migraine with aura
Source: Ann Clin Transl Neurol. 2020 Apr 21;7(5):855–9. doi: 10.1002/acn3.51040 (PMC7261755; doi:10.1002/acn3.51040)
Supplement: Supplementary file 1 — Data S1. Clinical features of family members. [file ACN3-7-855-s001.docx]

**A familial t(4;8) translocation segregates with juvenile myoclonic epilepsy, photosensitive occipital lobe epilepsy, and migraine with aura**

## *Supplementary file reporting the clinical features of family members.*

## The pedigree of the family is reported in Figure 1.

## The proband (IV:2) is a 22-year-old male who was born after 35 weeks of gestation with a dystocic deliver. He had delayed motor and cognitive milestones with a FSIQ 63, as assessed by the Wechsler Intelligence Scales for Children (WISC). At school learning difficulties were soon evident and so severe that he needed a support teacher. At the age of 8 years he experienced his first seizure, characterized by unresponsiveness, standing motionless for 1 min, then falling to the floor and vomiting. He had three similar critical episodes in the following 24 months. From the age of 10 years, he developed typical episodes of migraine with visual aura (MA). He also had stereotyped, transient episodes of elementary visual hallucinations that were not followed by headache or any other neurological symptoms.

Interictal awake and sleep EEGs showed bilateral occipital sharp-waves and sparse generalized spikes associated with both generalized and occipital photic-induced spikes. Brain MRI revealed a slightly larger cisterna magna. Valproate resolved all symptoms, and repeated EEGs were normal. At age 12 years, the medication was withdrawn, with no seizure recurrence. Brain MRI showed a slightly larger cisterna magna; cardiac evaluation with ECG and heart ultrasound revealed a right branch block and a mild tricuspid incompetence.

From the age 14 years, he developed weekly arm and leg jerks on awakening and sporadically during the day when relaxing. At that time, EEG revealed generalized 3.5-5 Hz spike-wave discharges with generalized paroxysmal responses. Moreover, at the age 14 years, the patient was noticed to be slower on cognition with anxiety, depression and obsessive-compulsive behavior. Lamotrigine (300 mg/day led to complete control of myoclonic seizures and repeated EEG recordings have been normal.

At the age of 17 years, he developed explosive and aggressive behavior that required treatment with risperidone with remission of symptoms. In the following months, however, because of the appearance of worrisome side effects, the most significant of which were increased appetite and extrapyramidal sings, the drug was replaced with quetiapine with partial improvement. The last neurological examination, at the age 22 years, disclosed bilateral hand tremor, brisk tendon reflexes on the four limbs, and flexor plantar responses. Visual acuity and fundoscopy were normal. Psychiatric examination revealed liability of mood and cognitive impairment, with failure to perform easy neuropsychological tasks. The results of a general physical examination revealed generalized obesity, short neck and head flexed on the trunk, fixed flexion of the left hand, elbows and knees. Dysmorphic features included short forehead, deep-set eyes, slant up palpebral fissures, large mouth, brachycephaly, prominent and low-set ears, cone-shaped fingers, flat arches of feet, that were kept extra-rotated, and hypoplastic toes nails. He could not stand, for instance to measure his weight. Impairments in standing and walking with frequent falls were documented in an orthopedic follow-up at the age of 15 years. Occipitofrontal circumference was 55.5 cm (50-75th centile), weight 75 kg (90th centile), and height 139 cm (<<3rd centile). Height of both parents were at the third centile; growth hormone deficiency was ruled out through appropriate dosages and tests.

The proband 24-year-old sister (III-1) had MA since adolescence. From age 13 years, she developed JME with daily myoclonic seizures exacerbated by stress, waking early and sitting too close to the TV. Generalized myoclonic-tonic-clonic seizures during sleep also occurred. Neurological examination and brain MRI were normal; interictal EEG showed bilateral occipital sharp-waves and generalized polyspike-waves with a generalized photoparoxysmal response. She was put on valproate that was substituted with levetiracetam with no further seizure; the last EEG was normal.

The 48-year-old proband’s father (II-2) had MA since age 11 years, but he has never had epileptic seizures. Neurological examination and brain MRI were normal; interictal awake and sleep EEG recordings showed bilateral occipital delta waves and rare generalized sharp-wave complexes during non-REM sleep. At follow-up, he has continued to have typical episodes of migraine with or without visual aura. He has recently been diagnosed to have a hypokinetic cardiomyopathy and a sigmoid aneurysm of interatrial septum.

The proband’s mother (II-1) and 19-year-old brother (III-3) did not experience any seizures or MA, and at the time of investigation their EEGs and brain MRI were normal. The paternal grandmother (I-1) is affected by a heart aneurysm of the left ventricle that was diagnosed at the age of 62 yeas. She gave birth to a dead male child (II-3). The proband’s maternal family history was unremarkable.
